# Impact of Short-Term Weight Loss on Hemostasis and Thrombosis after Bariatric Surgery

**DOI:** 10.1155/2023/1729167

**Published:** 2023-11-13

**Authors:** Ahmed Taha, Hasan Eroğlu, İskender Eren Demirbaş, Berkay Demir, Evren Dilektaşli

**Affiliations:** ^1^Yalova Training and Research Hospital, Department of General Surgery, Yalova, Türkiye; ^2^Bursa Kestel State Hospital, Department of General Surgery, Bursa, Türkiye; ^3^Malatya Darende Hulusi Efendi State Hospital, Department of General Surgery, Malatya, Türkiye; ^4^Bursa Yüksek İhtisas Training and Research Hospital Department of General Surgery, Bursa, Türkiye

## Abstract

**Introduction:**

Obesity causes thrombophilia and many coagulation problems related to slowing the capillary flow. We aimed to evaluate rapid weight loss outcomes in the early period after bariatric surgery on the coagulation system. *Materials and Method*. A prospective study enrolled 28 patients with a BMI > 40 kg/m^2^ who underwent bariatric surgery. Preoperative and postoperative (first and third months) demographic criteria—such as age, gender, weight, height, and alcohol and tobacco use, and biochemical parameters such as PLT, PT, aPTT, INR, bleeding time, coagulation time, fibrinogen, D-dimer, albumin, calcium, ionized calcium, vitamin D, and PTH—were analyzed.

**Results:**

We found that both bleeding and thrombotic parameters increase in early-slowing surgery. The first-month platelet levels were significantly different from the preoperative values (*p* < 0.001). The prothrombin time in the first (*p* < 0.001) and third months (*p* < 0.009) was also comparable. The PTT in the first month was higher than in the preoperative period (*p* < 0.011). INR in the first month (*p* < 0.001) was higher than that in the preoperative period and the third month (*p* = 0.007) value was higher than in the first month. In terms of fibrinogen levels, all parameters indicated statistical significance within each other; preoperative to the first month (*p* < 0.001), the first month to the third month (*p* < 0.016). Third-month D-dimer levels were lower than the first month's values (*p* = 0.032).

**Conclusion:**

Thromboembolic events have crucial importance in the converse scenario of haemorrhagic diathesis during the first months of bariatric surgery. Vitamin support and antithrombotic agents may be recommended in the early postoperative period.

## 1. Introduction

### 1.1. Background

Metabolic disorders such as deficiency of vitamins and electrolyte imbalance may cause coagulation problems after bariatric surgery. In the early postsurgical period, depletion of vitamin K-dependent coagulation factors may increase the risk of haemorrhage.

### 1.2. Aim and Objectives

We aimed to evaluate rapid weight loss outcomes in the early period after bariatric surgery on the coagulation system.

Today, mankind has been dealing with obesity all over the globe. It has become an epidemic because of fast food, which is cheap and easy to find, insufficient exercise, and a sedentary life [1–3]. Obesity is one of the important problems in public health due to its increasing prevalence, especially in industrialized countries [4, 5].

According to the World Health Organization (WHO) data, it has been reported that an increase of 10–30% has been detected in the prevalence of obesity in the last 10 years [6]. According to the data of the Ministry of Health and the preliminary study report of “Turkey Nutrition and Health Survey 2010,” the prevalence of obesity in Turkey was found to be 20.5% in men, 41.0% in women, and 30.3% in total [7].

Environmental factors and socioeconomic conditions such as behavioural disorders (overeating and lack of physical activity) increase an individual's risk of obesity [8]. In addition to environmental factors, some studies show that 40–70% of weight differences in humans can be determined by genetic factors [9, 10]. In addition, the main risk factors for the development of obesity are age, female gender, low education level, marriage, number of births, excess weight gained during pregnancy, use of oral contraceptives, early periods of smoking cessation, and alcohol intake [11, 12].

In its studies, The Organisation for Economic Co-operation and Development (OECD) indicates the danger of increased obesity and determines that one out of every five children in member countries is overweight or obese with an acceleration of obesity. Obesity increases considerably in adults, resulting in economic problems and premature deaths. It also causes serious health problems in new generations with its progressive onset in children at an early age [13]. By the way, the inadequacy of diet, exercise, and medical treatment, which has been utilized for many years in the struggle against obesity, and in addition to this, regaining the lost weight increases the popularity of surgical treatment day by day [2, 14].

Large-scale studies have been conducted due to the positive results of various bariatric surgery operations, which have increased significantly in recent years and are performed under the name of bariatric surgery [2]. With bariatric surgery, long-term permanent weight loss can be achieved, and many comorbidities are prevented by ameliorating the metabolic effects of obesity, thereby increasing the survival. Sustainable weight loss can be achieved with the highest rate of bariatric surgery, and more than 50% of excess weight can be lost [15].

Individuals who will undergo bariatric surgery are considered risky patients. Therefore, to achieve successful postoperative results, extensive evaluations before and after surgery are as important as the surgical technique. According to studies, obese patients are more frequently exposed to thromboembolic events [16, 17]. Weight loss and a decrease in body mass index (BMI) in patients undergoing bariatric surgery are expected to improve thromboembolic events [18].

On the other hand, metabolic diseases, vitamin deficiencies, and electrolyte disorders that develop after bariatric surgery may cause disorders in the coagulation system [5]. Subcutaneous ecchymoses and petechiae that may develop in the postoperative period are the clinical signs of this coagulation disorder [19].

### 1.3. Study Hypothesis

Following bariatric surgery, metabolic disorders such as deficiency of vitamins and electrolyte imbalance may cause coagulation problems. In the early postsurgical period, depletion of some vitamin K-dependent coagulation factors may increase the risk of haemorrhage; thus, anticoagulant agents may increase the thromboembolic event rate.

The primary endpoint of this study was to examine the weight loss in the early period in patients who underwent bariatric surgery, and the secondary endpoint was to investigate whether there were significant changes in bleeding and coagulation parameters in the early period with weight loss in the first and third months after surgery. We aimed to evaluate rapid weight loss outcomes in the early period after bariatric surgery on the coagulation system.

## 2. Materials and Methods

### 2.1. Study Design

A prospective study aimed to evaluate rapid weight loss outcomes in the early period after bariatric surgery on the coagulation system.

### 2.2. Study Setting and Population

A total of 28 individuals who applied to Bursa Yüksek İhtisas Training and Research Hospital in Turkey's General Surgery outpatient clinic due to morbid obesity have been included in the study. In order for the study to be performed simultaneously by different surgeons in the same center, patients who were compatible, did not have comorbidities, or were not taking medication were preferred. Suitable patients were selected within 5 months, and the operations were performed within 1 month. Postoperative follow-up lasted 4 months, and the study was completed in nearly 1 year.

### 2.3. Ethical Considerations

Ethics committee approval has been granted on protocol number 2011-KAEK-2015/18-02. Informed consent has been obtained from all the patients before the initiation of the study, and the study has been conducted within all the regulations of the declaration of Helsinki.

### 2.4. Inclusion Criteria

- Patients with a BMI > 40 kg/m^2^ who underwent surgery were prospectively included in the study.

### 2.5. Exclusion Criteria

- Subjects who did not volunteer to participate in the study,
- İndividuals who had a history of fibrinolytic, antithrombotic, anticoagulant drug (Aspirin, Coumadin) use,
- Previous deep vein thrombosis (DVT) or thromboembolic surgery was excluded from the study.

### 2.6. Variables

The following data were recorded in the preoperative (just before the surgery) and postoperative 1st and 3rd months in patients who underwent bariatric surgery: age, gender, weight, height, alcohol-smoking use, laboratory results (PLT, PT, aPTT, INR, bleeding time, clotting time, fibrinogen, D-dimer, albumin, calcium, ionized calcium, osteocalcin, vitamin D, and parathormone). In our preoperative clinic, routine blood count and detailed blood analysis, hormone analysis, coagulation tests, pulmonary function test, electrocardiogram, chest X-ray, gastroscopy, endocrinology consultation, psychiatry consultation, cardiology consultation and, if necessary, dietitian consultation are performed.

Demographic information (age, gender, weight, and height) of eligible patients and smoking and alcohol habits were recorded. Body mass index (BMI) was calculated by dividing the body weight in kilograms (kg) by the square of the height in meters (m). The ideal BMI was 25 kg/m^2^, and the ideal weight was taken as the average of the metropolitan index. Excess weight (EW) was calculated by subtracting the ideal weight from the measured patient weight. Percent excess weight loss (% EWL) and excess body mass index loss (% EBMIL) were calculated according to the following formulas:

- Percent loss of excess weight (% EWL) = (Preop. weight − postoperative weight × 100/excess weight)
- Percent excess BMI loss (% EBMIL) = (Preop. BMI − postoperative BMI × 100/(preoperative BMI − 25)).

### 2.7. Data Resource and Measurement

Bleeding and clotting times were evaluated manually. Hemogram and platelet count after taking into appropriate tubes (Mindray BC-6800 Auto Hematology Analyzer, Germany), measurement of hormones (Advia Centaur XP, USA), biochemistry values (Mindray BS 2000 Biochemistry Analyzer, Germany), and D-dimer (AQT90 flex, Denmark) devices. It was investigated whether there were significant changes in bleeding and coagulation parameters with the recorded weight loss. The data from 28 patients obtained were analyzed.

### 2.8. Statistical Methods

Statistical analysis was conducted in the SPSS 22.0 package program. The eligibility of the obtained data was interpreted with the Shapiro–Wilk test for normal distribution. Descriptive statistics for continuous variables were given as mean ± standard deviation for those showing a normal distribution and the median (minimum–maximum) for those not showing a normal distribution.

In the comparison of dependent groups, the paired samples “*t*” test was used for those conforming to the normal distribution, and the Wilcoxon signed-rank test was used for those not conforming to the normal distribution. The statistical significance was accepted as a *p* value of <0.05.

## 3. Results

### 3.1. Sociodemographic Analysis

A total of 31 patients have been operated and reviewed prospectively, but 3 patients were excluded because they did not meet the inclusion criteria. Twenty-five (89.3%) of the operated patients included in the study were female. The mean age of the patients was 37.68 years. The smoking rate has been found at 28.6% and alcohol consumption at 10.7%. Demographic characteristics were given in (Table 1).

### 3.2. Weight Loss Analysis

The first-month % EWL and % EBMIL scores were 21.7% (ranging between 13.0 and 45.6%) and 25.2% (13.8–49.7%), respectively. In the third month, % EWL and % EBMIL scores were 40.6% (ranging between 27.1 and 67.5%) and 45.5% (ranging between 29.4 and 76.6%). Postoperative weight follow-ups were given in Table 2. The success of the bariatric operation has been elaborated with the weight loss in the first month compared to the top preoperative period (*p* < 0.001), the decrease in BMI in the first month compared to the top preoperative period (*p* < 0.001), the weight loss in the third month compared to the preoperative period (*p* < 0.001), the decrease in BMI in the third month compared to the top preoperative period (*p* < 0.001), the weight loss in the third month compared to the first month (*p* < 0.001), and decrease in BMI in the third month compared to the first month.

### 3.3. Multivariate Analysis

The first-month platelet levels were significantly different from the preoperative values (*p* < 0.001). The prothrombin time in the first (*p* < 0.001) and third months (*p* < 0.009) was also comparable. The PTT in the first month was higher than in the preoperative period (*p* < 0.011). During the analysis of INR the first-month value (*p* < 0.001) was higher than the preoperative period and the third month (*p* = 0.007) value was higher than the first month. In terms of fibrinogen levels, all parameters indicated statistical significance within each other; preoperative to the first month (*p* < 0.001), the first month to the third month (*p* < 0.016). Third-month D-dimer levels were lower than the first month's values (*p* = 0.032). These parameters are elaborated in Table 3.

## 4. Discussion

Weight loss following bariatric surgery was evaluated by various methods. Total weight loss compared to the patient's weight before surgery, decrease in BMI, weight loss according to the standard weight and BMI, loss of excess weight relative to the standard weight (EWL) based on age and height, or loss in excess BMI based on BMI 25 kg/m^2^ (EBMIL) are some of these assessment methods [20]. The rate of weight loss (% EWL), which has been popularly used recently, showed a success target of 50 and above in the first 1-2 years [21]. In our study, % EWL and % EBMIL achieved in the first 3 months were calculated as 40.6 kg/m^2^ and 45.5 kg/m^2^, respectively, and no study in the literature has interpreted weight loss in the first 3 months. The previously published data focused on the 1-year results.

By homeostasis, there exists a delicate balance between coagulation and fibrinolysis in the vascular system, and the disruption of this balance leads to pathological events. The increase in obesity in coronary heart diseases, peripheral vascular diseases, stroke, and arterial and venous thrombosis [22] shows that this balance increases in favour of coagulation. Various clinical and epidemiological studies argue that there is a strong link between obesity and thrombosis [23, 24].

Increased fat mass in obesity is not only an increased fat tissue energy store but also an increase in the secretion of a metabolically active fat cell and an increased autocrine, paracrine, and endocrine effect. Leptin secreted from fat cells, adiponectin, resistin, plasminogen activator inhibitor-1 (PAI-1), tissue factor (TF), tumour necrosis factor-alpha (TNF-*α*), transforming growth factor-beta (TGF-*β*), and interleukin-6 (IL-6) all play an active role in thrombosis [18]. Increasing leptin in obese people causes an increase in insulin resistance and increases the incidence of stroke and myocardial infarction. Hyperfibrinogenemia found especially in obese women increases the risk of developing coronary and peripheral artery diseases, stroke, and venous thrombosis. Increased fibrinogen indicates the risk of developing arterial and venous thrombosis with fibrin formation, platelet aggregation, deterioration of blood viscosity, and atherosclerosis [25].

In recent years, many studies have been carried out, especially on TF, factor VII, and PAI-1. PAI-1, a serine protease inhibitor and one of the most important coagulating agents providing balance against the fibrinolytic system, has been shown to have a significant effect on the increase in morbidly obese patients and the increase in thrombotic events in obesity [26]. Along with it, an increase is detected in FVII, thrombin, thrombin-antithrombin complex (TAT), and TF activities, which have a significant role in thrombotic events [25, 26].

There are different and sometimes contradictory studies in the literature on the role of thrombocytes in obesity. The most important risk factors for increased venous thromboembolism in obese patients are inflammation, decreased fibrinolysis, increased thrombin formation, and increased platelet activation [17].

A significant improvement has been detected in morbidly obese patients in haematological changes in blood flow, such as increased blood and plasma viscosity [26]. Morbidly obese patients with either a low-calorie diet [27] or weight loss after bariatric surgery operations had an improvement in increased erythrocyte aggregation that appears in morbid obesity and impairs blood flow, and an improvement in the impaired lipid profile in hyperlipidemia has also been found [24]. Weight loss is associated with bariatric surgery and thromboembolic mediators such as PAI-1 and TF reduction, chronic inflammation, and metabolic changes, and provides an improvement in platelet dysfunction [27]. There are many studies in the literature investigating the individual and social benefits of long-term weight loss achieved with bariatric surgery in terms of socioeconomics and health in general. We wanted to emphasize that obesity is a thrombosis risk factor that can change and improve [18, 28].

Lupoli et al. reported improvement in impaired fibrinolytic activity in patients after bariatric surgery. In this study, while they recorded a 20% decrease in PAI-1 in the first months, they found a 10% decrease in t-PA. However, decreases were found in FVII, Protein C, and S [29]. These factors were related to vitamin K. The rapidly decreasing fat mass with weight loss may also be the cause of the fat-soluble vitamin K deficiency, and it also explains the increased bleeding risk in the early period [29]. The decrease in PAI-1 was found to be between 75 and 80% at 12 months in other studies. In this study by Ferrer et al., it was determined that the platelet volume, not the platelet count, changed in the postoperative 12th month. However, significant PT, CT prolongation, and fibrinogen change were also noted [30].

Pulmonary embolism (1%) and deep vein thrombosis from bariatric surgery and deep vein thrombosis (DVT) (1%) are significant causes of mortality. In a comprehensive 8-year review of 4293 patients at the Cleveland Clinic by Carmody et al., they found that laparoscopic bariatric surgery and/or postoperative prophylactic anticoagulant therapy did not change the risk of DVT. However, this study was only valid until the 24th post-operative day. In the study, they showed that conversion, increased age, and high BMI increased this risk [31]. In the study of Carmody et al., although routine heparin prophylaxis does not reduce this rate, the authors recommend more aggressive prophylaxis [32].

Mineral and vitamin deficiencies caused by rapid weight loss have been discussed in the literature [33]. In order to focus on the effectiveness of the study, we insisted on taking multivitamins to compensate for the possible vitamin deficiency that occurs during obesity surgery. As we stated in our study, patients were discharged with a multivitamin supplement that did not contain vitamin K in the postoperative period. The significant decrease in fibrinogen and platelet in the 1st month we recorded may be associated with an increase in consumptive coagulopathy and accompanying enhanced microembolic events. Prolongation in PT and aPTT and elevation in INR may suggest that intrinsic and extrinsic coagulation pathways are affected. These changes may have resulted from the decrease in coagulation factors that depend on hemostasis parameters. The increase in D-dimer in the 3rd month compared to the 1st month may be due to the partial decrease in consumption.

When these results are evaluated together, in the middle and long term of bariatric surgery there is improvement in hypercoagulopathy, but in the short term, an increase in bleeding diathesis due to some factor deficiencies and an increase in thromboembolic events due to various postoperative reasons are detected.

According to the different data, we recorded in our study and obtained from the literature, bleeding diathesis is also important along with thromboembolic events in the first months of bariatric surgery. The increase in the PT, aPTT, and INR values in the first and third months compared to the preoperative values, which we stated in the results of our study, was thought to indicate the bleeding diathesis occurring in this period. At the same time, considering the increase in D-dimer values and the subsequent regression in the third month, the risk of early bleeding and simultaneous thromboembolism can be considered in these patients. Therefore, it is important to carry out close follow-ups and laboratory screenings regularly in the first months [34]. Particular attention should be paid to bleeding that may occur in the stapler line after surgery. It may be recommended to use low-molecular-weight heparin (LMWH) for a long time in the early postoperative period together with vitamin support [35, 36].

### 4.1. Limitation of the Study

The study's main limitation could be attributed to comorbidities. Some of the patients included in the study used antihypertensive and antidiabetic drugs. It is not known whether these drugs will have a different effect on bleeding and coagulation parameters.

The study was performed at a designated time interval, in a single center with the same devices. The patients were followed up closely during the preoperative and postoperative periods. Individuals with fibrinolytic, antithrombotic, and anticoagulant use, previous deep vein thrombosis (DVT), or thromboembolic surgery were excluded from the study.

## 5. Conclusion

As obesity rates increase, the effectiveness of surgical treatment is obvious. Researching every aspect of obesity surgery increases the success of the treatment. New findings on this subject reduce possible complications. It can enlighten us about the precautions that can be taken to reduce the risk of clotting and bleeding that we have introduced. Therefore, new, prospective, randomized, large-scale case studies on bariatric surgery are needed.

## Figures and Tables

**Table 1:** Baseline demographics of the study population.

Gender (female), (%)	25 (89.3)
Age, mean ± SD (years)	37.68 ± 9.69
Height, median (min-max) (cm)	160 (150–188)
Preoperative weight, mean ± SD (kg)	123.38 ± 20.50
Smoking	8 (28.6)
Alcohol consumption	3 (10.7)

**Table 2:** Loss of excess weight and body mass index in the first and third months.

	End of first month	End of third month
% EWL, median (min-max)	21.7 (13.0–45.6)	25.2 (13.8–497)
% BMIL, median (min-max)	40.6 (27.1–67.5)	45.5 (29.4–76.6)

**Table 3:** Comparison of the treatment group parameters with preoperative values.

	(*n* = 28)	Pairwise comparisons	
Preoperative kg (Mean ± SD)	123.38 ± 20.50	Preoperative kg–First month kg	*p* < 0.001^*∗*^
First month kg (Mean ± SD)	109.07 ± 18.62	Preoperative kg–Third month kg	*p* < 0.001^*∗*^
Third month kg (Mean ± SD)	97.77 ± 15.68	First month kg–Third month kg	*p* < 0.001^*∗*^
Preoperative BMI (Mean ± SD)	46.86 ± 5.70	Preoperative BMI–First month BMI	*p* < 0.001^*∗*^
First month BMI (Mean ± SD)	41.46 ± 5.66	Preoperative BMI–Third month BMI	*p* < 0.001^*∗*^
Third month BMI (Mean ± SD)	37.26 ± 5.05	First month kg–Third month BMI	*p* < 0.001^*∗*^
Preoperative PLT median (min-max)	286 (128–393)	Preoperative PLT–First month PLT	*p* < 0.001^#^
First month PLT Median (min-max)	216 (139–349)	Preoperative PLT–Third month PLT	ns^#^
Third month PLT Median (min-max)	262.5 (155–460)	First month PLT–Third month PLT	0.001
Preoperative PT (Mean ± SD)	12.37 ± 1.07	Preoperative PT–First month PT	ns^*∗*^
First month PT (Mean ± SD)	12.53 ± 0.71	Preoperative PT–Third month PT	0.001^*∗*^
Third month PT (Mean ± SD)	13.10 ± 0.94	First month PT–Third month PT	0.009^*∗*^
Preoperative PTT (Mean ± SD)	32.26 ± 3.80	Preoperative PTT–First month PTT	0.025^*∗*^
First month PTT (Mean ± SD)	34.86 ± 5.46	Preoperative PTT–Third month PTT	0.011^*∗*^
Third month PTT (Mean ± SD)	35.24 ± 4.93	First month–Third month PTT	ns^*∗*^
Preoperative INR (Mean ± SD)	0.98 ± 0.10	Preoperative INR–First month INR	ns^*∗*^
First month INR (Mean ± SD)	1.01 ± 0.07	Preoperative INR–Third month INR	*p* < 0.001^*∗*^
Third month INR (Mean ± SD)	1.06 ± 0.09	First month INR–Third month INR	0.007^*∗*^
Preoperative BT (Mean ± SD)	82.25 ± 15.39	Preoperative BT–First month BT	ns^*∗*^
First month BT (Mean ± SD)	77.56 ± 11.94	Preoperative BT–Third month BT	ns^*∗*^
Third month BT (Mean ± SD)	83.07 ± 15.75	First month BT–Third month BT	ns^*∗*^
Preoperative CT median (min–max)	300 (4–361)	Preoperative CT–First month CT	ns^#^
First month CT median (min–max)	326.50 (280–400)	Preoperative CT–Third month CT	ns^#^
Third month CT Median (min–max)	340 (240–450)	First month CT–Third month CT	ns^#^
Preoperative FIB (Mean ± SD)	365.36 ± 55.60	Preoperative FIB–First month FIB	0.001^*∗*^
First month FIB (Mean ± SD)	313.01 ± 42.30	Preoperative FIB–Third month FIB	0.016^*∗*^
Third month FIB (Mean ± SD)	325.90 ± 47.35	First month FIB–Third month FIB	ns^*∗*^
Preoperative D-dimer median (min–max)	409 (267–1170)	Preoperative D-dimer–First month D-dimer	ns^#^
First-month D-dimer median (min–max)	489.5 (211–4530)	Preoperative D-dimer–Third month D-dimer	ns^#^
Third-month D-dimer median (min–max)	409 (162–1170)	First month D-dimer–Third month D-dimer	0.032^#^

^*∗*^Paired samples “*t*” test; ^#^Wilcoxon signed-rank test.

## Data Availability

The data used to support the findings of the study are available upon request to the corresponding author.

## Abbreviations

aPTT: Activated partial thromboplastin clotting time
BMI: Body mass index
DVT: Deep vein thrombosis
EBMIL: Excess body mass index loss
EW: Excess weight
IL-6: Interleukin-6
INR: International normalized ratio
OECD: Economic Assistance and Development Organization
PAI-1: Plasminogen activator inhibitor-1
PT: Prothrombin time
SPSS: Statistics Package for the Social Sciences
TAT: Thrombin-antithrombin complex
TE: Thromboembolic events
TF: Tissue factor
TGF-*β*: Transforming growth factor-beta
TNF-*α*: Tumour necrosis factor-alpha
WHO: World Health Organization
CT: Coagulation time
BT: Bleeding time.

## Additional Points

*What Is Already Known About This Topic?* (i) We know that obesity is a disease that causes thrombophilia and many problems related to the slowing of the capillary flow. (ii) Bariatric surgery causes rapid weight loss in the first months. (iii) Both overweight and rapid weight loss can cause metabolic problems. *What This Study Adds?* (i) We found that both bleeding and thrombotic events increase in the early time of bariatric surgery. (ii) The risk of bleeding and clotting continues in the first months of bariatric surgery. (iii) In the first months, it would be appropriate to take adequate precautions for both.
